# Apparent diffusion coefficient of vertebral haemangiomas allows differentiation from malignant focal deposits in whole-body diffusion-weighted MRI

**DOI:** 10.1007/s00330-017-5079-2

**Published:** 2017-11-13

**Authors:** Jessica M. Winfield, Gabriele Poillucci, Matthew D. Blackledge, David J. Collins, Vallari Shah, Nina Tunariu, Martin F. Kaiser, Christina Messiou

**Affiliations:** 1 Cancer Research UK Cancer Imaging Centre, Division of Radiotherapy and Imaging, The Institute of Cancer Research, 123 Old Brompton Road, London, SW7 3RP UK; 2 Department of Radiology, MRI Unit, The Royal Marsden NHS Foundation Trust, Downs Road, Sutton, Surrey, SM2 5PT UK; 3 Haemato-Oncology Research Unit, Division of Molecular Pathology, The Institute of Cancer Research, London, UK; 4 Department of Haematology, The Royal Marsden NHS Foundation Trust, Downs Road, Sutton, Surrey, SM2 5PT UK

**Keywords:** Diffusion magnetic resonance imaging, Whole body imaging, Bone neoplasms, Multiple myeloma, Haemangioma

## Abstract

**Objectives:**

The aim of this study was to identify apparent diffusion coefficient (ADC) values for typical haemangiomas in the spine and to compare them with active malignant focal deposits.

**Methods:**

This was a retrospective single-institution study. Whole-body magnetic resonance imaging (MRI) scans of 106 successive patients with active multiple myeloma, metastatic prostate or breast cancer were analysed. ADC values of typical vertebral haemangiomas and malignant focal deposits were recorded.

**Results:**

The ADC of haemangiomas (72 ROIs, median ADC 1,085×10^-6^mm^2^s^-1^, interquartile range 927–1,295×10^-6^mm^2^s^-1^) was significantly higher than the ADC of malignant focal deposits (97 ROIs, median ADC 682×10^-6^mm^2^s^-1^, interquartile range 583–781×10^-6^mm^2^s^-1^) with a p-value < 10^-6^. Receiver operating characteristic (ROC) analysis produced an area under the curve of 0.93. An ADC threshold of 872×10^-6^mm^2^s^-1^ separated haemangiomas from malignant focal deposits with a sensitivity of 84.7 % and specificity of 91.8 %.

**Conclusions:**

ADC values of classical vertebral haemangiomas are significantly higher than malignant focal deposits. The high ADC of vertebral haemangiomas allows them to be distinguished visually and quantitatively from active sites of disease, which show restricted diffusion.

***Key Points*:**

*• Whole-body diffusion-weighted MRI is becoming widely used in myeloma and bone metastases.*

*• ADC values of vertebral haemangiomas are significantly higher than malignant focal deposits.*

*• High ADCs of haemangiomas allows them to be distinguished from active disease.*

## Introduction

Vertebral haemangiomas are the most common benign vertebral neoplasm with a reported incidence of 10–26 % and are multiple in 7.2 % of normal subjects [1]. They are most commonly asymptomatic, although rarely (0.9–1.2 % [2]) lesions can cause symptoms through local mass effect, fracture or bleeding [3]. Histologically vertebral haemangiomas are composed of newly formed blood vessels usually with normal structure in the absence of arteriovenous shunts. Haemangiomas are not separated from surrounding bone by a capsule and the surrounding osseous lamellae usually shows secondary osteolysis and osteocondensation, and the bone marrow undergoes fibrous and/or adipose involution [2].

The variable composition of vertebral haemangiomas does lead to some variation in MRI appearances but vertebral haemangiomas usually display classical MRI appearances. On T_2_-weighted (T_2_w) images typical haemangiomas return high-signal intensity due to slow flow in vascular channels and oedema. Fat content results in high signal on T_1_-weighted (T_1_w) imaging. The presence of high signal on T_1_w or T_2_w images is related to the amount of adipocytes or vessels and interstitial oedema, respectively [4, 5]. Thickened trabeculae can sometimes be seen as linear low-signal intensity on all sequences [6]. As such, haemangiomas rarely present a diagnostic dilemma on routine MRI of the spine. However, applications for whole-body MRI are rapidly expanding and in particular whole-body MRI has become standard of care for myeloma and bone metastasis imaging in a growing number of institutions [7–9]. The complement of MR sequences used varies between institutions. For some applications such as screening for soft tissue malignancy in high-risk populations, whole-body MRI may consist of whole-body diffusion-weighted MRI without T_1_w and T_2_w imaging of the spine. In the absence of standard MRI sequences, vertebral haemangiomas may therefore be mistaken for sinister focal deposits.

Furthermore, healthcare systems with limited resource will struggle to maximise the potential of whole-body diffusion-weighted MRI. Automated segmentations made possible by machine learning technologies offer potential to not only speed up reporting times but also to transform whole-body qualitative data into quantitative datasets of disease burden and response to treatment [10]. Arguably the most exciting opportunities for such applications are in imaging bone disease but quantitative measures to enable exclusion of common vertebral haemangiomas from automated segmentations are not yet available.

In our institution whole-body diffusion-weighted MRI is standard of care for imaging patients with myeloma and is also frequently used to assess metastatic bone disease in patients with prostate or breast cancer. The potential mechanical complications in our patient population, which include vertebral fractures and spinal cord compression, necessitate inclusion of sagittal T_1_w and T_2_w MRI of the spine within the whole-body MRI protocol. This study therefore aims to identify apparent diffusion coefficient (ADC) values for typical haemangiomas in the spine and to compare them with malignant focal deposits.

## Materials and methods

This was a retrospective single-institution study with local institutional review board approval.

## Subjects

Whole-body MRI scans of 106 successive patients (57 prostate, 44 myeloma, five breast; 80 males, 26 females; median age 67 years, range 31–89 years) with active multiple myeloma and focal deposits (as per International Myeloma Working Group criteria [11]) or metastatic bone disease from prostate and breast cancer (confirmed on T_1_w and T_2_w MRI and sequential imaging) were included.

## MRI technique

Using an Avanto 1.5 T system (Siemens, Erlangen, Germany) a whole-body study was achieved by the serial acquisition of contiguous body regions. All subjects were scanned supine with arms by their sides. Coil elements were positioned from skull vertex to knees. Sagittal T_1_w images (TR 590 ms, TE 11 ms, field of view (FOV) 400 mm, slice thickness 4 mm), and T_2_w images (TR 2,690 ms, TE 93 ms, FOV 400 mm, slice thickness 4 mm) were acquired, followed by axial diffusion-weighted sequences (single-shot double spin echo echo-planar technique with STIR fat suppression in free breathing). b-values of 50 and 900 s mm^-2^ were applied in three orthogonal directions and combined to provide isotropic trace images. Diffusion-weighted images were acquired in multiple contiguous stations of 50 slices per station (slice thickness 5 mm, no gap, FOV 430 mm, phase direction AP, parallel imaging (GRAPPA) factor 2, TR 14,800 ms, TE 66 ms, inversion time (TI) 180 ms, voxel size 2.9 mm × 2.9 mm × 5 mm, number of signal averages acquired 4, matrix 150×150, bandwidth 1,960 Hz per pixel). The scanner carrier frequency used for the most superior imaging station was applied for all other stations. The same shim gradient currents were applied for each station. Total acquisition time was 50 min.

## Image analysis

**Fig. 1:**
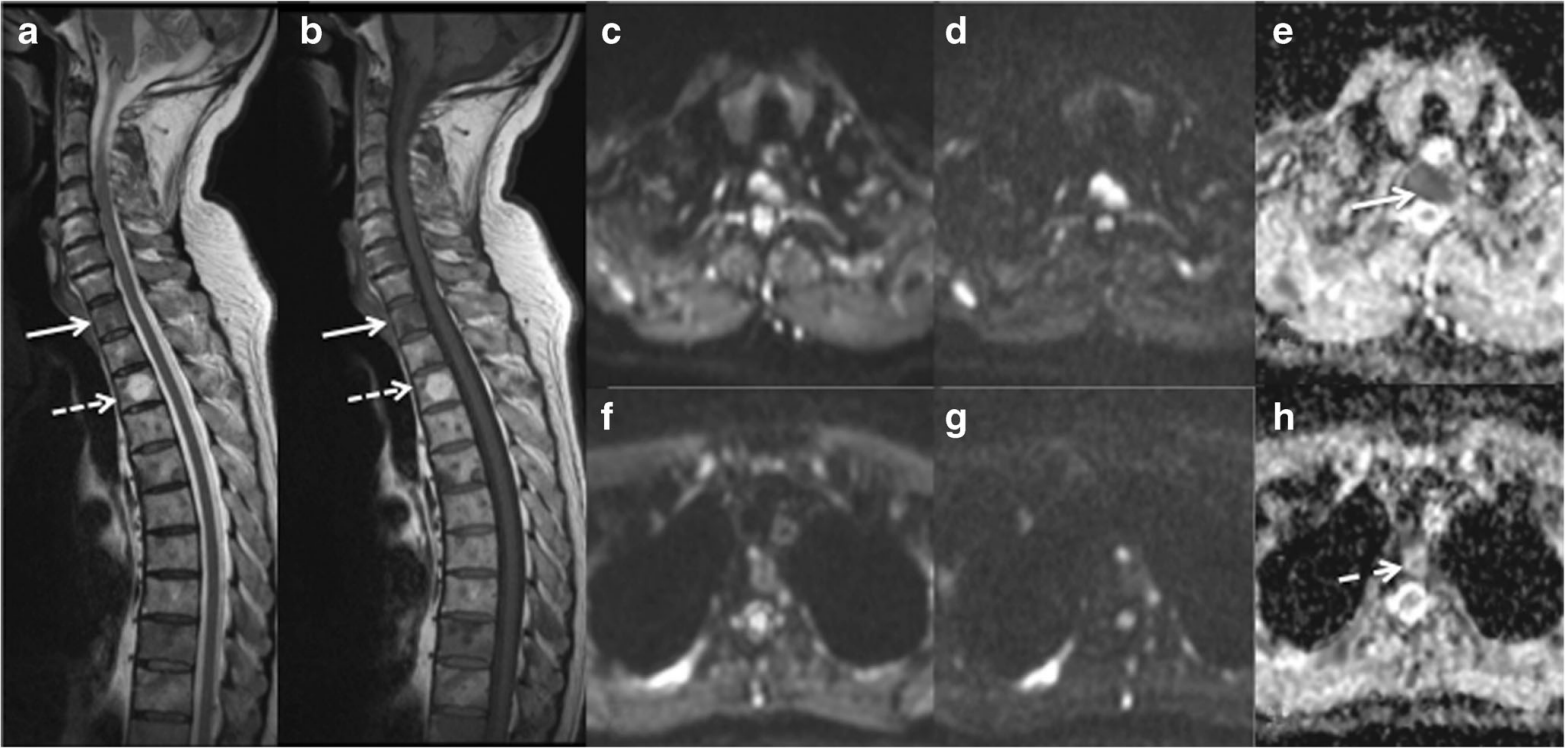
(**a**) Sagittal T_2_w and (**b**) T_1_w MRI of the cervico-thoracic spine shows a typical low signal metastasis (arrow) and a typical high signal haemangioma (dashed arrow). Corresponding axial diffusion-weighted MRI images of the metastasis (**c** b=50 s mm^-2^, **d** b=900 s mm^-2^, and **e** apparent diffusion coefficient (ADC) map) and of the haemangioma (**f** b=50 s mm^-2^, **g** b=900 s mm^-2^, and **h** ADC map) are shown. The metastasis exhibits restricted diffusion on the ADC map (**e**, arrow) but conversely the haemangioma has a high ADC (**h**, dashed arrow)

For each patient a maximum of three vertebral haemangiomas measuring a minimum diameter of 1 cm were identified as bright focal lesions on sagittal T_1_w and T_2_w imaging of the spine (Fig. 1). With the aid of the Picture Archiving and Communication System (PACS) localiser the corresponding lesion was identified on the axial ADC map, which was generated using a mono-exponential fit using the scanner’s proprietary software. A region of interest (ROI) was drawn around the haemangioma and the mean ADC calculated for all pixels within the ROI. For each patient a maximum of three malignant focal deposits measuring a minimum diameter of 1 cm were identified on axial b=900 s mm^-2^ diffusion-weighted images and ADC maps. An ROI was drawn around each deposit on the ADC map and the mean ADC calculated. The median diameter of ROIs was 18.6 mm (range 10.0–41.0 mm).

### Statistical analysis

Statistical analysis was carried out using Matlab version 2016a (The Mathworks, Natick, MA, USA). A two-sample t-test was used to assess whether there was a significant difference in ADC estimates between haemangiomas and malignant focal deposits. P<0.05 was considered to indicate a significant difference. Receiver operating characteristic (ROC) curves were used to assess the performance of ADC estimates in distinguishing haemangiomas from malignant focal deposits.

## Results

**Fig. 2:**
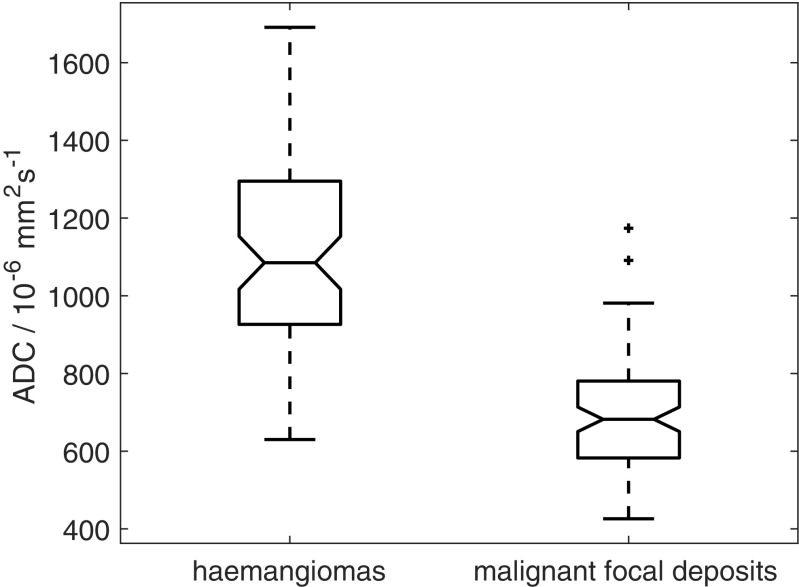
Boxplot showing all apparent diffusion coefficient (ADC) estimates from haemangiomas (n=72) and malignant focal deposits (n=97). Two-sample t-test, p<10^-6^

The ADC of haemangiomas (72 ROIs, median ADC 1,085×10^-6^mm^2^s^-1^, interquartile range 927–1,295×10^-6^mm^2^s^-1^) was significantly higher than the ADC of malignant focal deposits (97 ROIs, median ADC 682×10^-6^mm^2^s^-1^, interquartile range 583–781×10^-6^mm^2^s^-1^) with a p-value < 10^-6^ (Fig. 2).

**Fig. 3:**
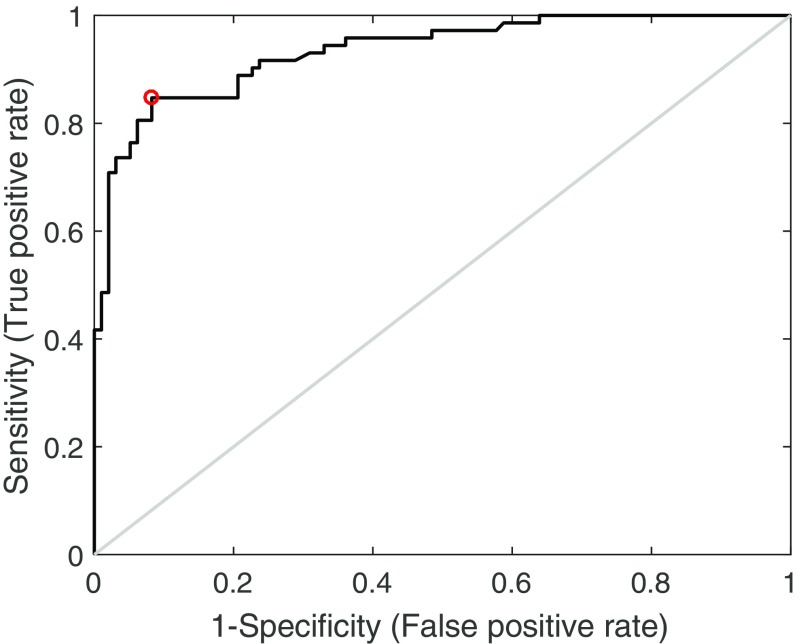
Receiver operating characteristic (ROC) curve showing performance of apparent diffusion coefficient (ADC) estimates in distinguishing haemangiomas from malignant focal deposits. Red circle shows optimal operating point. Grey line shows line of identity

ROC analysis produced an area under the curve of 0.93. An ADC threshold of 872×10^-6^mm^2^s^-1^ separated haemangiomas from malignant focal deposits with a sensitivity of 84.7 % and specificity of 91.8 % (Fig. 3).

## Discussion

This study demonstrated that ADC values of classical vertebral haemangiomas are significantly different to malignant focal deposits. The high ADC of vertebral haemangiomas allows them to be distinguished visually and quantitatively from active sites of disease, which show restricted diffusion. The ADC of malignant focal deposits (682×10^-6^mm^2^s^-1^) was in agreement with previously published values of 761×10^-6^mm^2^s^-1^ for myeloma [12] and 782×10^-6^mm^2^s^-1^ for bone metastases [13]. However, previous studies have reported ADC values of 960×10^-6^mm^2^s^-1^ following treatment [12] and therefore treated sites of disease could potentially be mistaken for haemangiomas in the absence of corresponding T_1_w and T_2_w imaging or previous imaging for comparison.

The ADC values of vertebral haemangiomas and malignant focal deposits in the present study are higher than ADC values of normal bone marrow reported in previous studies of healthy volunteers, which were acquired with comparable protocols [14, 15]. Mean ADCs of (471±142)×10^-6^mm^2^s^-1^ [14] and (577.4±56.9)×10^-6^mm^2^s^-1^ [15] have been reported in bone marrow in mixed cohorts of healthy volunteers of comparable ages to the patients in the present study. We were unable to confirm normal marrow for analysis in this cohort of patients as bone marrow in patients with myeloma or bone metastases may be abnormal even in the absence of focal lesions or clear evidence of diffuse signal abnormality.

The narrow range of ADC estimates observed across the 97 malignant focal deposits suggests that the whole-body diffusion-weighted MRI protocol employed in this study is robust and suitable for use in quantitative applications. The wider range of ADC estimates observed across the 72 haemangiomas may arise from variations in the composition of haemangiomas and may relate to differences in vascularity, fat content and oedema.

Accurate estimation of ADCs across the FOV is essential for the use of ADC thresholds to discriminate between imaging features, for example to distinguish haemangiomas from malignant focal deposits. Variation in ADC estimates has been demonstrated at points far from isocentre and has been attributed largely to non-linearity in the diffusion-encoding gradients [16, 17]. The variation in ADC estimates across the FOV in the whole-body diffusion-weighted MRI protocol employed in this study has been shown to be around 5–8 % but greater variations in ADC estimates would be encountered in imaging protocols that employ larger numbers of slices per station [17].

In conclusion, the ADC values of classical vertebral haemangiomas are significantly higher than the ADCs of malignant focal deposits. The high ADC of vertebral haemangiomas allows them to be distinguished visually and quantitatively from active sites of disease in whole-body diffusion-weighted imaging.

## Abbreviations

ADC: Apparent diffusion coefficient
AP: Anterior-posterior
FOV: Field of view
GRAPPA: Generalized Autocalibrating Partially Parallel Acquisitions
MRI: Magnetic resonance imaging
PACS: Picture Archiving and Communication System
ROC: Receiver operating characteristic
ROI: Region of interest
STIR: Short-TI inversion recovery
T_1_w: T_1_ weighted
T_2_w: T_2_ weighted
TE: Echo time
TI: Inversion time
TR: Repetition time
